# Association of Maternal Cervical Disease With Human Papillomavirus Vaccination Among Offspring

**DOI:** 10.1001/jamanetworkopen.2021.34566

**Published:** 2021-12-13

**Authors:** Christopher M. Worsham, Jaemin Woo, André Zimerman, Charles F. Bray, Anupam B. Jena

**Affiliations:** 1 Department of Health Care Policy, Harvard Medical School, Boston, Massachusetts; 2 Department of Medicine, Massachusetts General Hospital, Boston; 3 Division of Pulmonary and Critical Care Medicine, Massachusetts General Hospital, Boston; 4 Division of Cardiology, Hospital de Clinicas de Porto Alegre, Porto Alegre, Brazil; 5 Postgraduate Program in Cardiology and Cardiovascular Sciences, Federal University of Rio Grande do Sul, Porto Alegre, Brazil; 6 National Bureau of Economic Research, Cambridge, Massachusetts

## Abstract

**Importance:**

Barriers to childhood vaccination against vaccine-preventable diseases, such as those due to human papillomavirus (HPV), are well known. However, the role of salience bias—the change in perception of risk due to increased familiarity with the outcome—in decisions to vaccinate children has not been explicitly studied.

**Objective:**

To assess for salience bias in parental decisions to vaccinate children.

**Design, Setting, and Participants:**

This retrospective cohort study used a time-to-event (survival) analysis to compare vaccination rates of children whose mothers had a history of cervical cancer or a cervical biopsy, who have experienced adverse vaccine-preventable outcomes, and for whom vaccination may be more salient, with a control group of children whose mothers had no such history. Participants were accrued from the MarketScan Commercial Database, including US children who turned 11 years old, when HPV vaccination is recommended, from January 1, 2014, to December 31, 2018. Data were analyzed from December 29, 2020, to September 17, 2021.

**Exposures:**

Maternal history of cervical cancer or cervical biopsy.

**Main Outcomes and Measures:**

Vaccination against HPV.

**Results:**

A total of 757 428 children (370 878 girls [49.0%] and 386 550 boys [51.0%]) were identified, of whom 38 366 had mothers with a history of cervical biopsy alone and 1084 had mothers with a history of cervical cancer. Overall, 54.2% of children (55.7% of girls and 52.7% of boys) received at least 1 vaccination by 16 years of age. In a time-to-event analysis, HPV vaccination did not differ between children whose mothers had cervical cancer vs those whose mothers did not (hazard ratio [HR] for girls, 0.99 [95% CI, 0.86-1.13]; HR for boys, 1.08 [95% CI, 0.94-1.24]). Maternal history of cervical biopsy was associated with a minimally increased hazard of vaccination (HR for girls, 1.06 [95% CI, 1.04-1.09]; HR for boys, 1.04 [95% CI, 1.01-1.06]). There were no clinically meaningful differences between groups for the tetanus/diphtheria/acellular pertussis and meningococcal vaccinations, which are also recommended at 11 years of age.

**Conclusions and Relevance:**

In this analysis of salience bias in childhood vaccination decisions, mothers’ personal history of cervical cancer or cervical biopsy was not associated with greater vaccination rates among children against HPV. These findings suggest that salience of vaccine-preventable outcomes may not have a major impact on childhood vaccine hesitancy in HPV; the role of salience should be investigated for other vaccines.

## Key Points

**Question:**

Is salience bias—the change in perception of risk due to increased familiarity with the outcome—associated with human papillomavirus (HPV) vaccination decisions?

**Findings:**

In this cohort study of 757 428 children, there was no major difference in the HPV vaccination rate of children whose mothers had a history of either cervical cancer or a cervical biopsy compared with children whose mothers had neither history.

**Meaning:**

These findings suggest that salience of vaccine-preventable outcomes is not associated with childhood vaccine hesitancy.

## Teaser

This cross-sectional study assesses the potential role of salience in influencing vaccination decisions by analyzing human papillomavirus vaccination rates among preadolescent children whose mothers had a history of cervical cancer or cervical biopsy.

## Introduction

Many barriers to vaccinating populations against preventable disease are well known, including affordability, access, and misperceptions about vaccine efficacy and safety.^1,2,3,4,5^ However, hesitancy surrounding the COVID-19 vaccine^3,4,5^ has highlighted the need for better understanding of the potential motivators of vaccination decisions.

Although the US Centers for Disease Control and Prevention (CDC) recommends that nearly all adolescents receive several vaccines, vaccination rates often fall short of the goal. In 2017, for example, the CDC estimated that only 48.6% of adolescents were up to date on the human papillomavirus (HPV) vaccine, which prevents cervical cancer, although 65.5% had received at least 1 dose of the multidose recommended series (current recommendations are to initiate a 2-dose series for children aged 11-12 years; patients who initiate the series at 15 years or older require 3 doses).^6,7^ Although practical considerations—such as affordability and availability of vaccines—can be barriers to vaccination, psychological factors such as complacency about the pathogen and low confidence in the safety and efficacy of the vaccine contribute to vaccine underuse worldwide.^8^

Vaccination, and disease prevention more generally, can be particularly challenging because patients and populations often lack personal or emotional connections to the outcome being targeted.^9^ Parental decisions on whether to vaccinate their children might reasonably be influenced by how they perceive risk of the disease or risk of a devastating outcome—an outcome with which a parent may have no personal connection or experience. For example, parents who choose not to vaccinate their young children against diseases such as measles perceive their children as less susceptible to infection and perceive the disease as less severe than parents who fully vaccinate their children.^10^

However, when large outbreaks of a vaccine-preventable disease such as measles occur within a local community, rates of vaccination against that disease increase.^11^ This change is likely due to an increase in perceived risk by parents from a combination of an often small increase in absolute risk of the disease and a newfound connection with the disease. This increased perceived risk based solely on personal connection is an example of salience bias, or a tendency to judge the risk of an event by how personally connected one is to the outcome.

In a seminal 1974 study, Tversky and Kahneman framed salience bias in simple terms: “the impact of seeing a house burning on the subjective probability of such accidents is probably greater than reading about a fire in a local paper.”^12^^(p1127)^ Salience bias has been shown to affect decision-making in many areas both inside and outside of medicine, such as automobile pricing, energy conservation while showering, and preferences around cardiopulmonary resuscitation in advance care planning.^13,14,15^ In other areas of medicine, it is possible that a lack of disease salience may lead people to not seek recommended care.

We assessed the potential role of salience in influencing vaccination decisions by analyzing HPV vaccination rates among preadolescent children whose mothers had experienced adverse outcomes that can result from HPV infection (eg, cervical cancer) and are now vaccine preventable. We used a nationwide database of insurance claims to select children who turned 11 years old after the HPV vaccine was added to the recommended vaccine schedule and compared HPV vaccination rates between children whose mothers did vs did not have a history of cervical abnormalities (cervical cancer or need for cervical biopsy). We hypothesized that a large increase in HPV vaccination rates would occur owing to salience (eg, at least 10%) among children whose mothers had experience with cervical abnormalities compared with children whose mothers did not.

## Methods

### Data Sources

We used the MarketScan Commercial Database (IBM Corp), which contains commercial insurance claims of tens of millions of Americans covered by employer-sponsored insurance plans from more than 300 employers. This database has been used in prior studies of vaccinations, including the HPV vaccine, in children, adolescents, and adults.^16,17,18,19,20,21,22^ Owing to the use of deidentified data, this study was deemed exempt from review by the Harvard Medical School Institutional Review Board. The study followed the Strengthening the Reporting of Observational Studies in Epidemiology (STROBE) reporting guideline.

### Study Population

The primary study population was children who turned 11 years old between January 1, 2014, and December 31, 2018, who were continuously enrolled since their 9th birthday, and whose mothers and metropolitan statistical area (MSA) of residence were identifiable in the database. Children were followed up for as long as they were continuously enrolled after their 11th birthday; as such, children contributed variable person-time to the analyses.

The HPV vaccine has been recommended by the CDC’s Advisory Committee on Immunization Practices for routine vaccination of girls since 2006 and boys since 2011. We selected children who turned 11 years old starting in 2014 because by then the HPV vaccine would have been a well-established routine vaccination for both girls and boys. Because the Advisory Committee on Immunization Practices allows but does not specifically recommend vaccination as early as 9 years of age, we limited our sample to children who were followed up continuously in the database since that age in case they were vaccinated early, which may have been more likely among children of mothers with a history of cervical abnormalities.

### Outcome Measures

Human papillomavirus vaccination was identified by the presence of a billing code for an HPV vaccine administration, defined by *Common Procedural Terminology* (*CPT*) codes 90649, 90650, and 90651. The time elapsed was recorded in months from the month of the child’s 9th birthday to the date of vaccine administration. If a child received a second vaccine dose, the timing of that dose was also recorded. Falsification analyses were performed using alternate, non-HPV vaccinations, which were identified by procedure codes for a single dose of the meningococcal vaccine (identified by *CPT* codes 90619, 90620, 90621, 90644, 90733, and 90734) or the tetanus/diphtheria/acellular pertussis (Tdap) vaccine (*CPT* code 90715).

### Patient Characteristics and Covariates

Children’s month and year of birth were obtained from the database to determine when they turned 11 years of age, when the first HPV vaccination was recommended. Other child demographic characteristics, including sex, MSA of residence, state of residence, and number of insurance dependents in the child’s family, were also obtained from the database. Mean per-capita income and percentage of adults with a bachelor’s degree or higher level of education in 2011 for the primary insurance holder’s MSA of residence were obtained from the US Bureau of Economic Analysis^23^ and the US Census Bureau,^24^ respectively. The mean number of preventive care visits per year was determined by the number of unique insurance claims for ambulatory encounters with *CPT* codes 99381 to 99387 or 99391 to 99397, divided by the number of years the participant was included in the database.

### Maternal Covariates

Participants’ mothers were identified as the female primary policy holder or the female spouse of a male primary policy holder of the insurance policy under which the child was listed as a dependent. We determined maternal history based on diagnosis codes from mothers’ insurance claims before the first of the following events: the child’s first HPV vaccination, turning 16 years of age, or the child’s departure from the database. Mothers were classified into 3 mutually exclusive groups: (1) history of cervical cancer; (2) history of cervical biopsy without a history of cancer; or (3) controls without a history of cervical biopsy or cervical cancer based on insurance claims.

History of cervical cancer was defined by *International Classification of Diseases, Ninth Revision*, codes 180.X for claims before October 1, 2015, and *International Statistical Classification of Diseases and Related Health Problems, Tenth Revision*, codes C53.X for subsequent claims. History of cervical biopsy was defined by *CPT* codes 57452, 57454, 57455, 57456, 57460, and 57461.

### Statistical Analyses

Data were analyzed from December 29, 2020, to September 17, 2021. Baseline characteristics between groups of children whose mothers had a history of cervical abnormalities were compared with those of controls using standardized mean differences^25^ (values <0.1 were considered not to be clinically significant). We then compared vaccination rates of children in a retrospective cohort study using a cumulative time-to-event (survival) analysis, stratified by both the child’s sex and mother’s history of cervical abnormalities. We hypothesized that mothers with a history of cervical abnormalities would be more likely to have their children vaccinated and more likely to vaccinate them sooner owing to personal history with cervical disease, which would increase salience of HPV vaccination. Although the CDC recommends HPV vaccination among children aged 11 and 12 years, it can be given to children as young as 9 years and to adolescents of any age. A cumulative time-to-event analysis was therefore an ideal approach to detect differences between strata of maternal cervical history during a long period with variable follow-up time among the children studied.

In each time-to-event analysis, participants were followed up from their ninth birthday (the earliest the CDC suggests the vaccine can be given) until the outcome of interest (primary analysis, first HPV vaccination; events for secondary analyses included the participant’s second HPV vaccination, first Tdap vaccination, or first meningococcal vaccination), or until a censoring event, including departure from the database or the month of their 16th birthday at the end of the study period. The study period ended at the 16th birthday to allow any delayed vaccinations to be captured while maximizing the sample size of continuously enrolled patients. We stratified by the child’s sex because we hypothesized a larger effect of salience on vaccination of girls, who are at risk for adverse cervical outcomes. We used Kaplan-Meier methods to generate cumulative event curves and tested differences between strata using the log-rank test.

We conducted a Cox proportional hazards regression analysis to address the possibility that unmeasured confounders might lead to a lack of observed evidence of maternal salience in child HPV vaccinations. Specifically, if confounding factors (eg, socioeconomic factors) are associated with greater likelihood of maternal cervical abnormalities and lower rates of child HPV vaccinations, a failure to account for these factors would lead us to spuriously conclude that a maternal history of cervical abnormalities has no salience for child HPV vaccinations (ie, it does not raise the likelihood of vaccination). We used Cox proportional hazards regression to generate models that were adjusted for state of residence, calendar month of birth (which has been previously associated with variation in child vaccination rates^19^), year of birth, number of insurance dependents in the household, number of child preventive care visits, MSA mean per capita income, and MSA percentage of adults with a bachelor’s degree or higher level of education.

We conducted several additional analyses. First, because attitudes toward HPV vaccination may have changed over time, we conducted the same analyses stratified by the years in which the child turned 11 years old (2012-2014 vs 2015-2016). Second, we conducted similar time-to-vaccination analyses for non-HPV vaccinations recommended at 11 years of age (meningococcal and Tdap vaccines) under the assumption that any unobserved confounding factors in the HPV analysis would likely lead to differences in non-HPV vaccination rates between children whose mothers had varying cervical abnormality histories, a falsification analysis. Third, to assess for the possibility of bias introduced by static group assignment, we repeated the time to first HPV vaccination analysis excluding children whose mothers’ cervical biopsy or cervical cancer diagnosis occurred after the child turned 11 years of age.

Analyses were performed using R, version 3.5.2 (R Program for Statistical Computing), and STATA, version 15 (StataCorp LLC). The 95% CIs around estimates reflect an α level of .025 in each tail. Two-sided *P* < .05 indicated statistical significance.

## Results

### Study Population

The primary study population included 757 428 children, of whom 370 878 (49.0%) were girls and 386 550 (51.0%) were boys. Overall, 38 366 children (5.1% of total; 18 738 girls and 19 628 boys) had mothers with a history of cervical biopsy alone, 1084 children (0.1% of total; 542 boys and 542 girls) had mothers with a history of cervical cancer, and 717 978 children (94.8% of total; 351 598 girls and 366 380 boys) had mothers without a known history of cervical biopsy or cervical cancer. Characteristics of these groups were similar (Table 1; eTable 1 in the Supplement provides the standardized mean differences comparing characteristics for each pair of groups, all of which were <0.1 except for small differences in the number of insurance dependents in the child’s family).

**Table 1.** Characteristics of the Study Population^a^

Characteristic	Girls (n = 370 878)				Boys (n = 386 550)	
	Controls	Mothers with cervical abnormalities requiring biopsy	Mothers with cervical cancer	Controls	Mothers with cervical abnormalities requiring biopsy	Mothers with cervical cancer
No. (%) of total by sex	351 598 (94.8)	18 738 (5.1)	542 (0.1)	366 380 (94.8)	19 628 (5.1)	542 (0.1)
Year of ninth birthday						
2012	92 124 (26.2)	3999 (21.3)	123 (22.7)	96 293 (26.3)	4422 (22.5)	142 (26.2)
2013	66 550 (18.9)	3434 (18.3)	94 (17.3)	68 999 (18.8)	3650 (18.6)	106 (19.5)
2014	70 486 (20.0)	3992 (21.3)	124 (22.9)	73 417 (20.0)	4178 (21.3)	114 (21.0)
2015	65 167 (18.5)	3882 (20.7)	99 (18.3)	67 559 (18.4)	3896 (19.8)	91 (16.8)
2016	57 271 (16.3)	3431 (18.3)	102 (18.8)	60 112 (16.4)	3482 (17.7)	89 (16.4)
No. of annual preventive care visits, mean (SD)	0.52 (0.35)	0.51 (0.34)	0.51 (0.35)	0.53 (0.35)	0.52 (0.35)	0.50 (0.35)
No. of dependents in family						
1	45 649 (13.0)	3144 (16.8)	86 (15.9)	45 300 (12.4)	3212 (16.4)	89 (16.4)
2	164 374 (46.8)	8676 (46.3)	249 (45.9)	171 800 (46.9)	9189 (46.8)	256 (47.2)
3	94 793 (27.0)	4665 (24.9)	131 (24.2)	100 058 (27.3)	4976 (25.4)	133 (24.5)
4	32 598 (9.3)	1625 (8.7)	39 (7.2)	34 225 (9.3)	1596 (8.1)	42 (7.7)
≥5	14 184 (4.0)	628 (3.3)	37 (6.8)	14 997 (4.1)	655 (3.3)	22 (4.1)
MSA per-capita income, mean (SD), natural logarithm $	10.7 (0.19)	10.7 (0.19)	10.7 (0.19)	10.7 (0.19)	10.7 (0.19)	10.7 (0.19)
Bachelor’s degree or higher level of education, mean (SD), % in MSA	30.7 (6.75)	30.6 (6.46)	30.5 (6.66)	30.7 (6.76)	30.5 (6.47)	30.3 (6.75)

Abbreviation: MSA, metropolitan statistical area.

^a^ Unless otherwise indicated, data are expressed as number (%) of children in each group. Percentages have been rounded and may not total 100. Standardized differences between groups within each sex are provided in eTable 1 in the Supplement.

### First Dose of HPV Vaccination

Among 281 823 children who were followed up until the month of their 13th birthday or beyond (138 001 girls and 143 822 boys), for whom at least 1 HPV vaccination should have been performed under CDC guidelines, only 54.2% (55.7% of girls and 52.7% of boys) had received at least 1 vaccine by 16 years of age, findings consistent with national survey data.^6^

**Figure 1.**
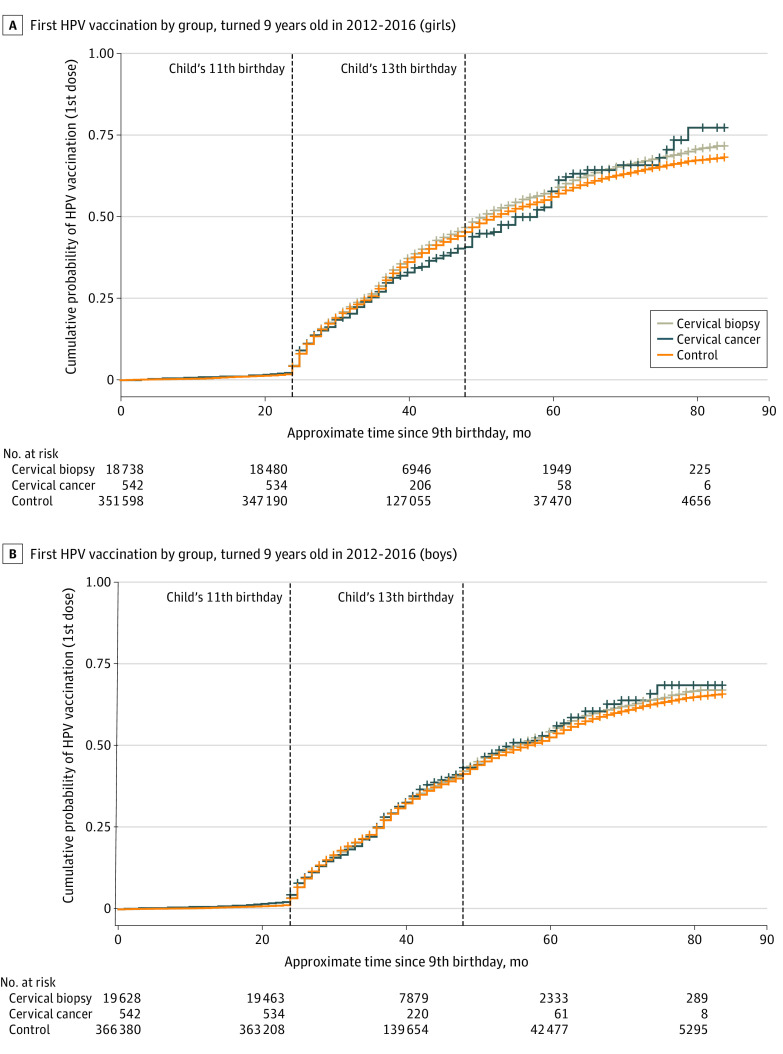
Kaplan-Meier Curves of Time to First Human Papillomavirus (HPV) Vaccine, Stratified by Sex and Maternal History of Cervical Cancer and Biopsy

For both boys and girls, HPV first-dose vaccination patterns were similar regardless of maternal cervical history. For example, unadjusted analysis of time to first dose (Kaplan-Meier analysis) for girls showed that although the timing of the first HPV vaccine dose was statistically significantly different between groups of girls based on maternal cervical history (log-rank χ^2^ = 15.8; *P* < .001), the differences were clinically small, particularly during the CDC-recommended vaccination window of 11 to 12 years of age (Figure 1). Similar findings were also observed after multivariable adjustment in the Cox proportional hazards model (Table 2), in which maternal history of cervical cancer was not associated with a difference in hazard of first-dose HPV vaccination (hazard ratio [HR], 0.99 [95% CI, 0.86-1.13]; *P* = .86) and maternal cervical biopsy was associated with only slightly higher hazard of first-dose vaccination (HR, 1.06 [95% CI, 1.04-1.09]; *P* < .001) when compared with girls without a maternal history of cervical cancer or cervical biopsy.

**Table 2.** Cox Proportional Hazards Regression Models^a^

Model	Girls		Boys	
	Adjusted HR (95% CI)	*P* value	Adjusted HR (95% CI)	*P* value
Time to first dose				
Control	1 [Reference]	NA	1 [Reference]	NA
Cervical biopsy	1.06 (1.04-1.09)	<.001	1.04 (1.01-1.06)	.002
Cervical cancer	0.99 (0.86-1.13)	.86	1.08 (0.94-1.24)	.27
Time to second dose				
Control	1 [Reference]	NA	1 [Reference]	NA
Cervical biopsy	1.05 (1.02-1.07)	<.001	1.03 (1.01-1.06)	.01
Cervical cancer	1.00 (0.87-1.15)	.99	1.08 (0.94-1.24)	.26

Abbreviations: HR, hazard ratio; NA, not applicable.

^a^ Models are adjusted for state of residence, birth month, birth year, number of dependents in household, mean number of annual preventive visits, metropolitan statistical area (MSA) mean per capita income, and MSA percentage with bachelor’s degree or higher level of education. Likelihood ratio, Wald test, and log-rank test *P* values were less than .01 for all models.

For boys, unadjusted analysis of time to first dose showed no patterns of statistically or clinically significant difference (log-rank χ^2^ = 3.6; *P* = .20) of first HPV vaccine dose for boys with varying maternal cervical history (Figure 1). Similar findings were again observed after multivariable adjustment in the Cox proportional hazards regression model (Table 2), in which boys with a maternal history of cervical cancer were no more likely to receive the first dose of the HPV vaccine (HR, 1.08 [95% CI, 0.94-1.24]; *P* = .27) and boys with a maternal history of cervical biopsy had only slightly higher hazard of first-dose HPV vaccination (HR, 1.04 [95% CI, 1.01-1.06]; *P* = .002) compared with boys without a maternal history of cervical cancer or cervical biopsy.

### Second Dose of HPV Vaccination

**Figure 2.**
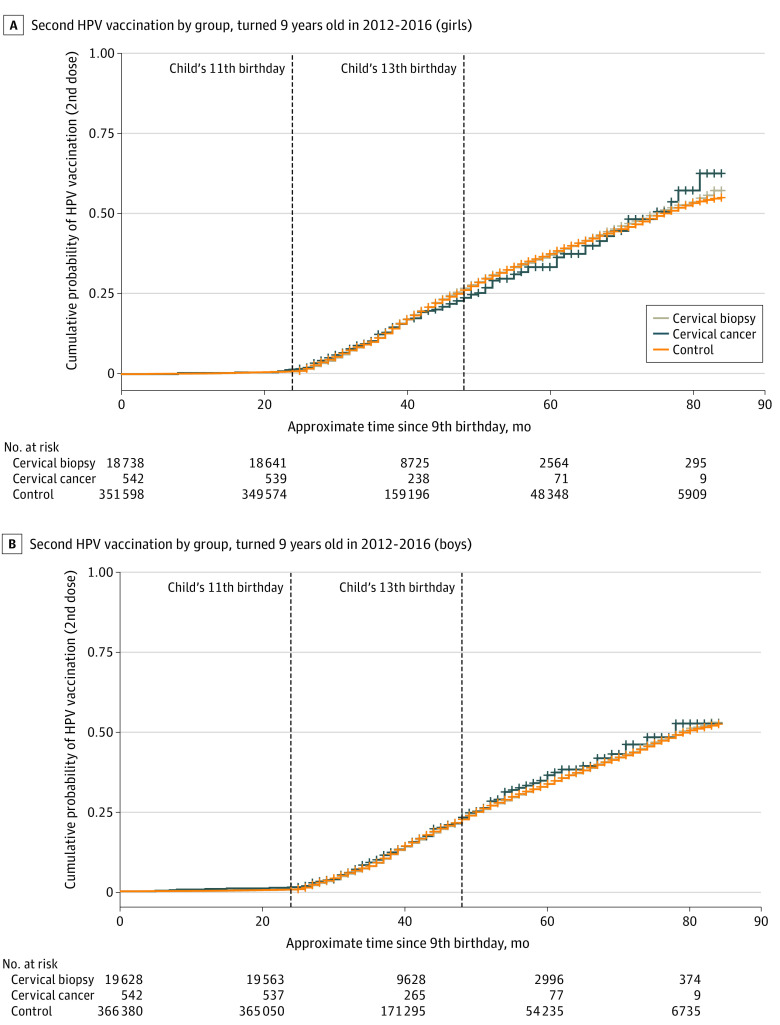
Kaplan-Meier Curves of Time to Second Human Papillomavirus (HPV) Vaccine, Stratified by Sex and Maternal History of Cervical Cancer and Biopsy

Among 281 823 children (138 001 girls and 143 822 boys) who were followed up until or beyond the month of their 13th birthday (the age by which CDC guidelines recommend at least 2 HPV vaccinations should have been administered on the routine schedule), only 38.3% (40.2% of girls and 36.5% of boys) had received 2 vaccines. Findings similar to those for analyses of time to first dose were observed in analyses of time to second dose comparing children whose mothers had a history of cervical cancer or cervical biopsy and those whose mothers did not; findings were similar for both boys (cervical cancer: HR, 1.08 [95% CI, 0.94-1.24]; *P* = .26 and cervical biopsy: HR, 1.03 [95% CI, 1.10-1.06]; *P* = .01) and girls (cervical cancer: HR, 1.00 [95% CI, 0.87-1.15]; *P* = .99 and cervical biopsy: HR, 1.05 [95% CI, 1.02-1.07]; *P* < .001) before and after adjustment (Figure 2 and Table 2).

### Additional Analyses

Additional analyses supported findings of the primary analysis. First, because attitudes toward HPV vaccines may have changed over time and their inclusion to the routine vaccination schedule for both boys and girls occurred in 2011, we repeated our analyses of time to first dose with stratifying according to the year in which a child turned 9 years of age (eFigure 1 in the Supplement). Similar vaccination patterns were observed by maternal cervical history for both boys and girls who turned 9 years of age between 2012 and 2014 vs 2015 and 2016. Second, to assess the possibility of unmeasured confounders that could be associated with child HPV vaccinations and maternal history of cervical abnormalities, we repeated the analysis of time to vaccination for both the meningococcal and Tdap vaccines via a falsification analysis (eFigures 2 and 3 and eTable 2 in the Supplement). As with the HPV vaccine, both the meningococcal and Tdap vaccines are recommended at 11 years of age according to the CDC schedule.^7^ There were no major differences in meningococcal or Tdap vaccination patterns or vaccination rates between girls and boys whose mothers varied in history of cervical cancer or biopsy. Third, to assess for the possibility of bias introduced by static group assignment, we repeated the analysis of time to first HPV vaccination, excluding children whose mothers received a cervical biopsy (889 girls and 910 boys) or were diagnosed with cervical cancer (32 girls and 30 boys) after the child turned 11 years of age (eFigure 4 in the Supplement); the vaccination pattern was unchanged.

## Discussion

In an analysis of approximately 750 000 children with commercial insurance in the US, a maternal history of cervical cancer or cervical abnormalities requiring biopsy was not associated with a major increase in vaccination against HPV in sons or daughters of vaccine-eligible age. These findings suggest that mothers’ personal experiences with cervical abnormalities, including cervical cancer, was not a salient enough factor to induce major increases in vaccination rates against HPV when families otherwise would not have been vaccinated.

The World Health Organization’s Strategic Advisory Group of Experts on Vaccine Hesitancy^8^ developed the “3 Cs” model of vaccine hesitancy, which places its many drivers into 3 problem categories: confidence, complacency, and convenience. Under this framework, there are several possible explanations for why families with mothers who had cervical cancer or cervical biopsies were not more likely to vaccinate their children against HPV. If families with a maternal history of cervical cancer or biopsy have not causally connected their diagnosis with HPV—which may be common^26,27^—then they may not have greater salience and thus no difference in complacency with respect to HPV vaccination for their children. Alternatively, families who have causally connected their diagnosis to HPV may not be more likely to vaccinate their children if the increased salience does not serve to adequately reduce complacency about the virus, overcome low confidence and mistrust in vaccines in general, or overcome attitudes specifically surrounding the HPV vaccine. Our finding that girls were only 3% more likely than boys to be vaccinated at all and no more likely to be vaccinated if the mother had a history of cervical cancer or biopsy further suggests that the risk of the preventable outcome is not the primary driver of HPV vaccination decisions.

Notably, in our study and in data collected annually by the CDC,^6^ HPV vaccination rates were lower than rates for the Tdap and meningococcal vaccines, which are recommended at the same age, suggesting unique barriers to HPV vaccination. One such factor may be stigma^28^ based on misconceptions that the HPV vaccine would lead children to more promiscuous sexual behavior, which fueled politicization^29^ of the vaccine around the time it was recommended for widespread use—not unlike recent politicization surrounding the COVID-19 vaccine.^3,4,5^ Importantly, 1 large-scale study of childhood HPV vaccination^30^ found that vaccination was not associated with any greater risk of sexually transmitted diseases. Further research is needed to understand the association between parental decision-making and personal experience with adverse outcomes, knowledge of their causative factors, and other factors such as political beliefs and group identity that may contribute more to vaccination decisions.

### Limitations

This study has several limitations. First, residual confounding by factors associated with both a mother’s history of cervical cancer or cervical biopsy and the family’s likelihood to vaccinate their child is possible, despite adjustments for family and regional characteristics. Vaccination rates and patterns were similar among groups in the Tdap and meningococcal vaccine analyses, which suggests that confounding factors do not explain the entire lack of observed evidence for major influence of salience in influencing HPV vaccinations specifically. Second, in addition to the assumption of proportional hazards between groups during the study period, the Cox proportional hazards regression models also assumed fixed group assignment, which could introduce bias. Although it is possible that a mother was diagnosed with cervical cancer during the child’s inclusion period—meaning that a child could have been assigned to the group with maternal history of cervical cancer even though until that time they were similar to another group—there is no evidence in our results of any significant bias introduced by these definitions. Third, our analysis was restricted to children whose mothers were on the same insurance policy, which allowed for family linkages; a *mother* was defined narrowly in this study owing to restrictions imposed by the nature of the claims data, and thus a child’s true mother(s) may not have been accurately identified in a small number of patients. However, given the high prevalence of HPV and its cervical complications among all maternal populations in the years included in this study, it is unclear why this restriction would lead to selection bias that would affect the study’s conclusions. Finally, this study was performed in a population of commercially insured children, limiting generalizability to other populations.

## Conclusions

The findings of this time-to-event cohort study suggest that children whose mothers had a history of cervical cancer or cervical abnormalities requiring biopsy were not more likely to vaccinate their children against HPV, despite their personal experience with vaccine-preventable adverse outcomes. These findings also suggest that mothers with a history of adverse cervical outcomes may be either unaware of the causal link between HPV and cervical disease or that from a behavioral perspective, increased salience of the risks of HPV does not outweigh other factors that contribute to vaccine hesitancy.

## Notes

Supplement. **eTable 1.** Standardized Mean Differences Between Groups by Sex **eTable 2.** Cox Proportional Hazards Regression Models for Tdap and Meningococcal Vaccination **eFigure 1.** Time to First HPV Dose Analyses, Stratified by Sex, Maternal Cervical History, and Year, Unadjusted **eFigure 2.** Time to Tdap Analyses, Stratified by Sex and Maternal Cervical History, Unadjusted **eFigure 3.** Time to Meningococcal Vaccine Analyses, Stratified by Sex and Maternal Cervical History, Unadjusted **eFigure 4.** Time to First HPV Vaccination Analyses, Excluding Children Whose Mothers Underwent Cervical Biopsy or Cervical Cancer Diagnoses After the Child Turned 11 Years Old
