# Supplementary material for: Association of Maternal Cervical Disease With Human Papillomavirus Vaccination Among Offspring
Source: JAMA Netw Open. 2021 Dec 13;4(12):e2134566. doi: 10.1001/jamanetworkopen.2021.34566 (PMC8669541; doi:10.1001/jamanetworkopen.2021.34566)
Supplement: Supplement. — eTable 1. Standardized Mean Differences Between Groups by Sex eTable 2. Cox Proportional Hazards Regression Models for Tdap and Meningococcal Vaccination eFigure 1. Time to First HPV Dose Analyses, Stratified by Sex, Maternal Cervical History, and Year, Unadjusted eFigure 2. Time to Tdap Analyses, Stratified by Sex and Maternal Cervical History, Unadjusted eFigure 3. Time to Meningococcal Vaccine Analyses, Stratified by Sex and Maternal Cervical History, Unadjusted eFigure 4. Time to First HPV Vaccination Analyses, Excluding Children Whose Mothers Underwent Cervical Biopsy or Cervical Cancer Diagnoses After the Child Turned 11 Years Old [file jamanetwopen-e2134566-s001.pdf]

Supplementary Online Content

Worsham CM, Woo J, Zimerman A, Bray CF, Jena AB. Association of maternal cervical disease with human papillomavirus vaccination among offspring. *JAMA Netw Open*. 2021;4(12):e2134566. doi:10.1001/jamanetworkopen.2021.34566

eTable 1. Standardized Mean Differences Between Groups by Sex

eTable 2. Cox Proportional Hazards Regression Models for Tdap and Meningococcal Vaccination

eFigure 1. Time to First HPV Dose Analyses, Stratified by Sex, Maternal Cervical History, and Year, Unadjusted

eFigure 2. Time to Tdap Analyses, Stratified by Sex and Maternal Cervical History, Unadjusted

eFigure 3. Time to Meningococcal Vaccine Analyses, Stratified by Sex and Maternal Cervical History, Unadjusted

eFigure 4. Time to First HPV Vaccination Analyses, Excluding Children Whose Mothers Underwent Cervical Biopsy or Cervical Cancer Diagnoses After the Child Turned 11 Years Old

This supplementary material has been provided by the authors to give readers additional information about their work.

© 2021 Worsham CM et al. *JAMA Network Open*.

eTable 1. Standardized Mean Differences Between Groups by Sex

	Female				Male		
	Control vs. Biopsy	Control vs. Cancer	Biopsy vs. Cancer		Control vs. Biopsy	Control vs. Cancer	Biopsy vs. Cancer
**Year of 9th Birthday**							
2012	0.11	0.08	0.03		0.09	<0.01	0.09
2013	0.02	0.04	0.03		<0.01	0.02	0.02
2014	0.03	0.07	0.04		0.03	0.03	<0.01
2015	0.06	<0.01	0.06		0.04	0.04	0.08
2016	0.05	0.07	0.01		0.04	<0.01	0.04
**Birth Month**							
January	0.02	0.09	0.07		0.04	0.03	0.06
February	<0.01	0.05	0.07		0.01	0.04	0.05
March	<0.01	<0.01	0.04		<0.01	<0.01	0.02
April	<0.01	0.02	<0.01		0.01	0.04	0.03
May	<0.01	0.02	0.02		<0.01	0.04	0.05
June	0.01	0.04	0.06		<0.01	0.01	0.01
July	<0.01	0.06	0.06		<0.01	0.01	0.01
August	<0.01	0.02	0.01		<0.01	0.01	<0.01
September	0.02	0.02	<0.01		0.03	0.06	0.09
October	0.01	0.04	0.04		<0.01	0.08	0.09
November	0.01	0.08	0.09		0.09	0.02	<0.01
December	<0.01	0.01	0.01		<0.01	0.03	0.04
**Annual Preventive Care Visits, mean**	0.03	0.03	<0.01		0.03	0.07	0.05
**Dependents in Family**							
1	0.11	0.08	0.03		0.11	0.12	<0.01
2	<0.01	0.02	<0.01		<0.01	<0.01	<0.01
3	0.05	0.06	0.02		0.04	0.06	0.02
4	0.02	0.08	0.06		0.04	0.06	0.01
5+	0.04	0.08	0.16		0.04	<0.01	0.04
**MSA Per Capita Income (natural log USD), mean**	<0.01	0.02	0.01		<0.01	0.03	0.02
**MSA Percentage with Bachelor's Degree or More, mean**	0.02	0.03	<0.01		0.03	0.06	0.04

Notes: MSA = metropolitan statistical area. USD = United States Dollars.

© 2021 Worsham CM et al. *JAMA Network Open*.

eTable 2. Cox Proportional Hazards Regression Models for Tdap and Meningococcal Vaccination

	**Females**			**Males**	
	Adjusted Hazard Ratio (95% CI)	p-value		Adjusted Hazard Ratio (95% CI)	p-value
	**Time-to-Tdap Vaccine Models**				
**Control**	ref			ref	
**Cervical Biopsy**	0.92 (0.90-0.94)	< 0.01		0.93 (0.91-0.94)	< 0.01
**Cervical Cancer**	0.83 (0.75-0.93)	< 0.01		0.89 (0.80-1.00)	0.05
	**Time-to-Meningococcal Vaccine Models**				
**Control**	ref			ref	
**Cervical Biopsy**	0.94 (0.92-0.96)	< 0.01		0.93 (0.91-0.95)	< 0.01
**Cervical Cancer**	0.81 (0.72-0.91)	< 0.01		0.87 (0.77-0.97)	0.01

Notes: Other adjustments include state of residence, birth month, birth year, number of dependents in household, mean number of annual preventive visits, MSA mean per capita income, MSA percentage with Bachelor's degree or more. Likelihood ratio, Wald, and log-rank test p-values were < 0.01 for all models.

© 2021 Worsham CM et al. *JAMA Network Open*.

eFigure 1 Time to First HPV Dose Analyses, Stratified by Sex, Maternal Cervical History, and Year, Unadjusted

(A) Turned 9 in 2012-2014

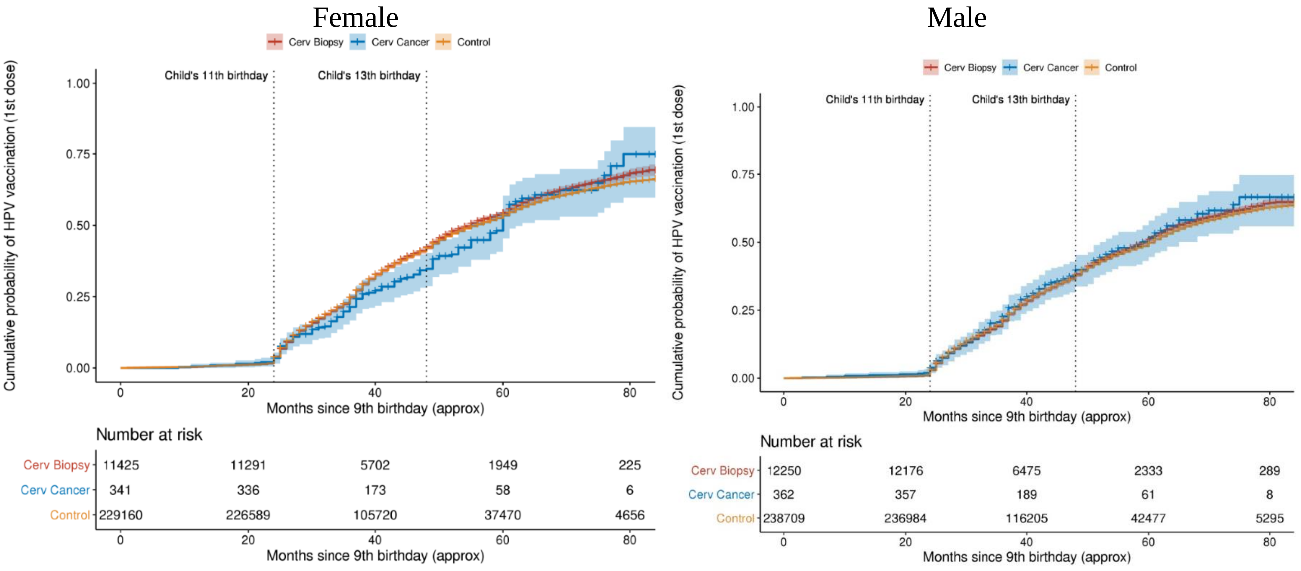

(B) Turned 9 in 2015-2016

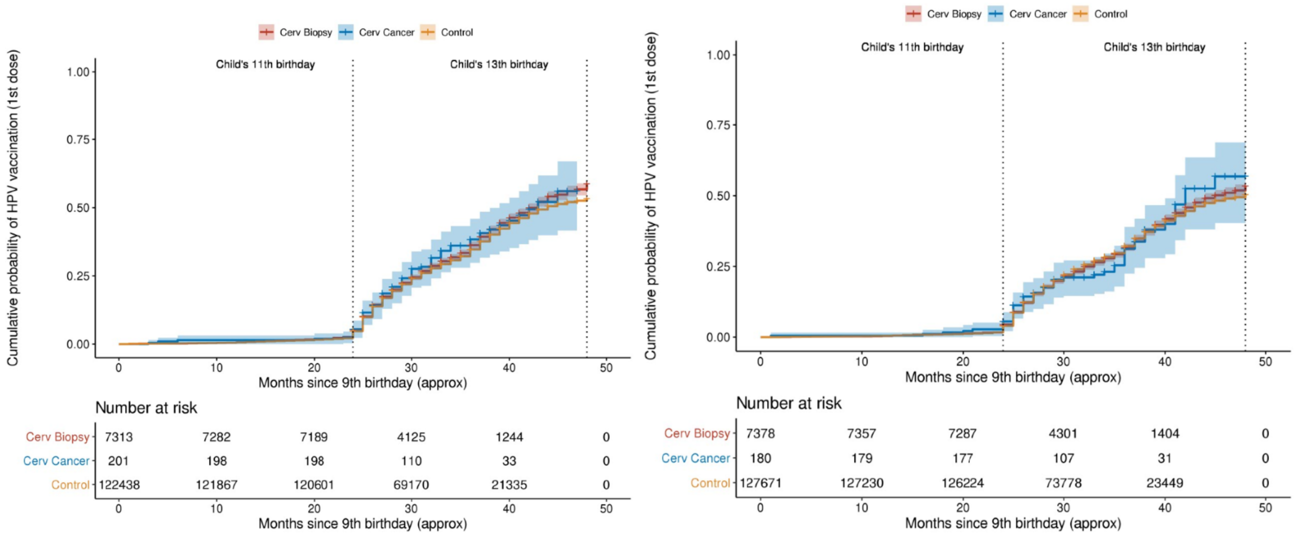

Notes: HPV = human papilloma virus.

© 2021 Worsham CM et al. *JAMA Network Open*.

eFigure 2. Time to Tdap Analyses, Stratified by Sex and Maternal Cervical History, Unadjusted

Female Male

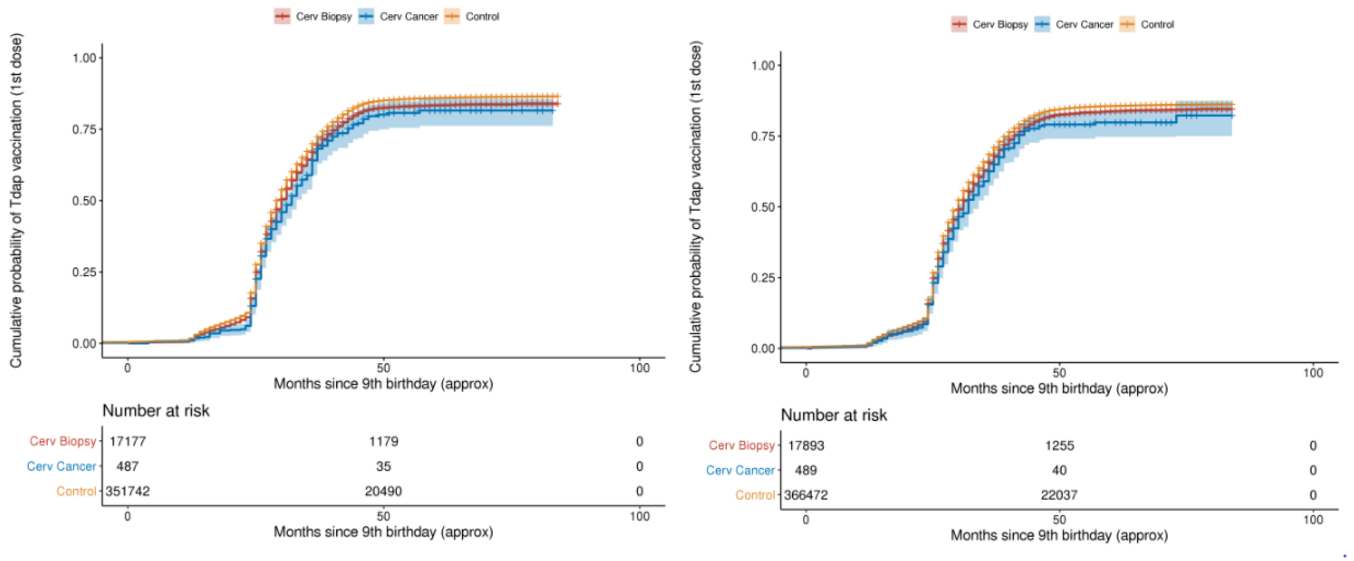

Notes: Tdap = tetanus, diphtheria, pertussis

© 2021 Worsham CM et al. *JAMA Network Open*.

eFigure 3. Time to Meningococcal Vaccine Analyses, Stratified by Sex and Maternal Cervical History, Unadjusted

Females | Males

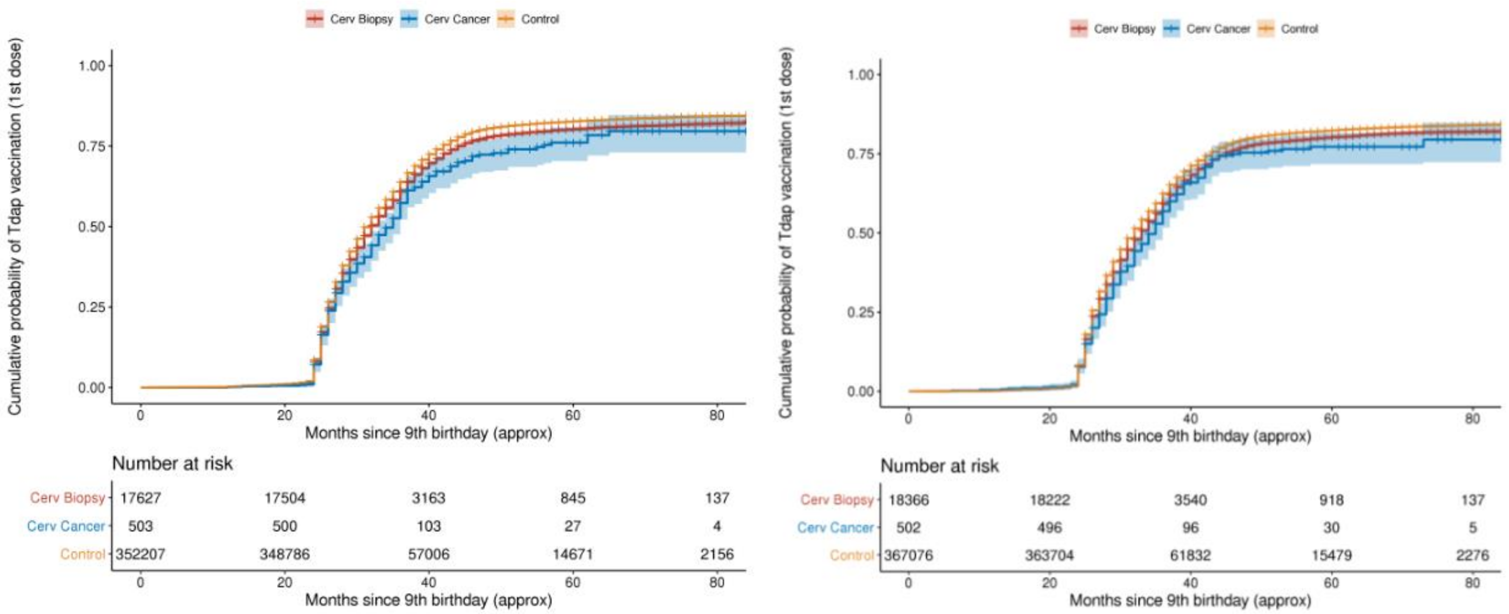

© 2021 Worsham CM et al. *JAMA Network Open*.

eFigure 4. Time to First HPV Vaccination Analyses, Excluding Children Whose Mothers Underwent Cervical Biopsy or Cervical Cancer Diagnoses After the Child Turned 11 Years Old

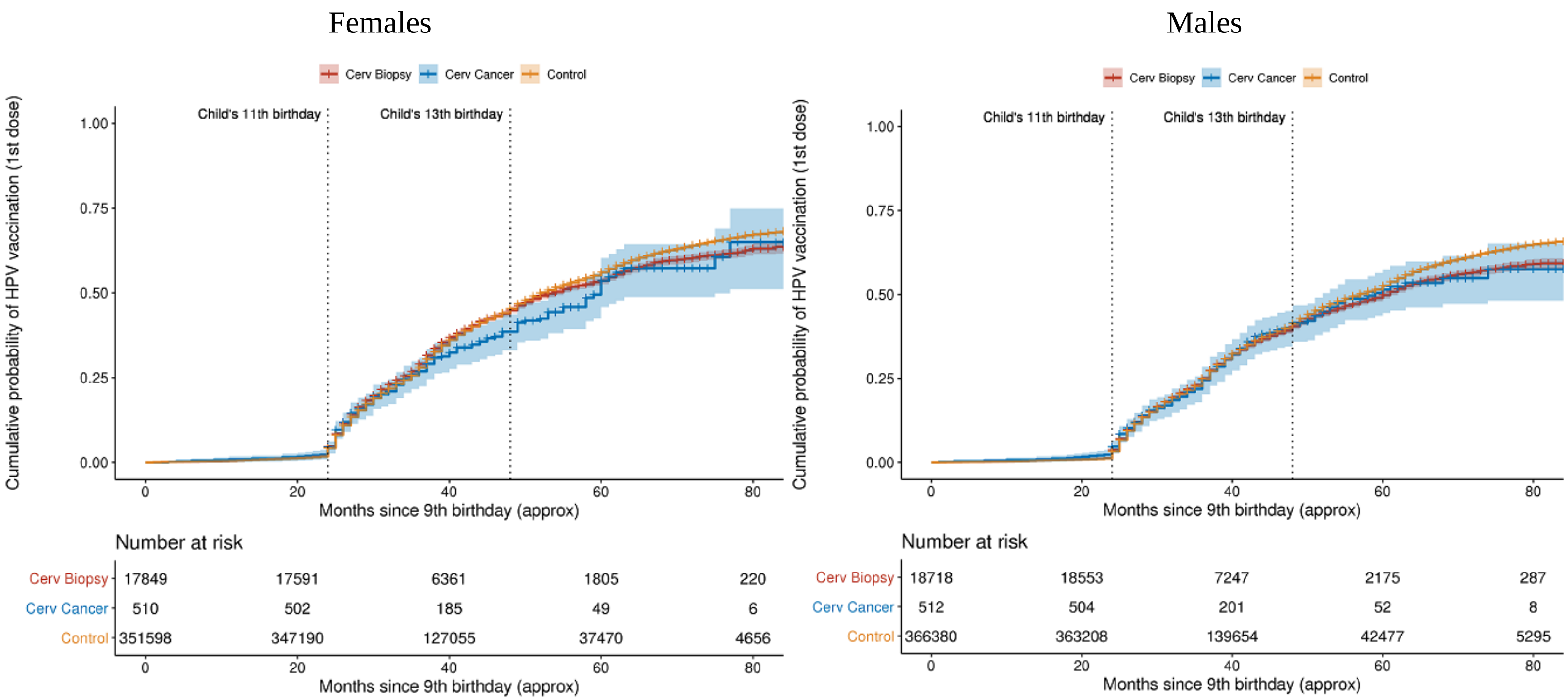

© 2021 Worsham CM et al. *JAMA Network Open*.
